# *MCP-1/CCR2* axis regulates M2 macrophage polarization and immunosuppression in mycobacterium tuberculosis

**DOI:** 10.3389/fimmu.2025.1698369

**Published:** 2026-01-05

**Authors:** Guizeng Zhao, Can Guo, Xiaoyang Li, Lei Tan, Xueyang Chen, Feng Tian, Lili Zhang, Xia Wang, Junwei Cui

**Affiliations:** 1 The First Affiliated Hospital of Xinxiang Medical University, Weihui, Xinxiang, China; 2 Department of Bacteriology and Immunology, Beijing Chest Hospital, Capital Medical University/Beijing Tuberculosis and Thoracic Tumor Research Institute, Beijing, China

**Keywords:** H37Ra, immunity, M2 macrophage polarization, MCP-1/CCR2 axis, mycobacterium tuberculosis

## Abstract

**Background:**

Mycobacterium tuberculosis (MTB) evades host immunity and maintains chronic infection, in part by reprogramming macrophage function. The chemokine *MCP-1* and its receptor *CCR2* play a key role in attracting monocytes and immunological modulation, but their exact involvement in MTB pathogenesis is unknown.

**Methods:**

Using the H37Ra-infected mouse model, the expression of MTB virulence marker ESAT-6 and autophagy marker Beclin-1 was assessed. Transcriptome analysis was performed to identify *CCR2*-related gene expression changes and enriched pathways. In addition, the effects of *CCR2* antagonists and *MCP-1* knockdown on macrophage apoptosis, polarization, cytokine production, and immunosuppressive signaling were assessed using Quantitative real-time PCR, ELISA, flow cytometry, immunohistochemistry, immunofluorescence, and western blot.

**Results:**

*CCR2* inhibition reduced ESAT-6 expression and restored Beclin-1 levels in lung tissue, alleviating inflammation and injury during late-stage infection. Transcriptomic profiling revealed that H37Ra infection activated *CCR2*-dependent genes involved in immune response and apoptosis, including *Trim30*, *Fas*, and *PD-1*, which were reversed by *CCR2* antagonists. At the cellular level, H37Ra upregulated *MCP-1* expression, promoting M2 polarization. *MCP-1* Knockdown enhanced macrophage apoptosis, reversed M2 polarization, and suppressed immunosuppressive signaling. Additionally, *MCP-1* knockdown increased TNF-α and IFN-γ levels, reduced TGF-β and IL-10 secretion, and oppositely regulated ESAT-6 and Beclin-1 expression.

**Conclusion:**

The *MCP-1/CCR2* axis promotes M2-type macrophage polarization, suppresses apoptosis, and enhances immunosuppressive signaling in the context of H37Ra infection. Targeting *CCR2/MCP-1* may provide a promising strategy to reverse immune evasion and restore host defense mechanisms during MTB infection.

## 1 Introduction

Tuberculosis is a contagious lung disease attributed to infection with Mycobacterium tuberculosis (MTB) (1). Patients with active tuberculosis release MTB into the air through droplets generated by coughing, sneezing, and other actions, and healthy individuals may become infected after inhaling these bacteria-laden droplets (2). Although the host immune response is crucial for controlling M. tuberculosis infection, persistent or latent infection remains challenging due to the complex immune evasion mechanisms of MTB. Macrophages are highly plastic immune cells that can polarize into classically activated M1 or activated M2 phenotypes depending on environmental cues. M1 macrophages primarily mediate antimicrobial and pro-inflammatory responses, whereas M2 macrophages facilitate tissue repair and immunosuppression. Recent studies highlight the relevance of macrophage polarization across various diseases (3, 4). Acting as frontline immune cells, alveolar macrophages limit bacterial dissemination through phagocytosis and the secretion of antimicrobial and pro-inflammatory factors (5). However, MTB can induce macrophage polarization to the M2 type, suppressing inflammatory responses and weakening phagocytic and bactericidal functions, thus creating an immunosuppressive environment that promotes its survival (6). Additionally, Early Secreted Antigenic Target-6 (ESAT-6), a key virulence factor secreted by MTB, can disrupt host cell membranes, interfere with autophagy, induce apoptosis in immune cells, and increase M2-related cytokine expression, which worsens pulmonary immune imbalance (7, 8). Therefore, understanding how MTB regulates macrophage polarization and virulence factors like ESAT-6 to mediate immunosuppression is essential for understanding its persistence and developing new treatments.

Monocyte chemoattractant protein-1 (*MCP-1*, also referred to as *CCL2*) and the chemokine receptor *CCR2* are important mediators in the control of tissue homeostasis, inflammatory responses, and monocyte movement (9). *CCR2* is mainly expressed on the surface of myeloid cells such as monocytes and macrophages, mediating their recruitment to inflammatory sites (10). *MCP-1* is the main chemokine secreted by a variety of cells under infection or stimulation (11). Previous research has demonstrated that the *CCR2/MCP-1* signaling axis plays a core immune regulatory function in a variety of pathological conditions. For example, a study (12) showed that MTB antigens such as PPD and ManLAM can selectively downregulate the expression of *CCR2* in antigen-presenting cells without affecting *CCR5* or *CCR7*, suggesting that *CCR2* has a specific regulatory role in MTB-induced immune responses. ManLAM can also induce the production of *MCP-1/CCL2*, which may be related to IFN-γ signaling. This highlights the *CCR2/MCP-1* axis’s regulating function in the immunological milieu of MTB infection. Similarly, in a mouse lung adenocarcinoma model, *Ccr2/MCP-1* promotes the accumulation of myeloid suppressor cells (MDSCs), enhances local immunosuppression, as well as promotes tumor progression (13). Therefore, the *Ccr2/MCP-1* signaling axis contributes to the remodeling of the disease microenvironment and immune escape by regulating immune cell migration and local inflammatory response.

Despite its known involvement in immune regulation, the specific function of the *Ccr2*/MCP-1 axis in MTB infection is yet to be fully elucidated. In order to explore the immunoregulatory role of the *Ccr2/MCP-1* signaling axis in the process of MTB infection, the H37Ra chronic tuberculosis mouse model and *in vitro* macrophage infection system were established. The effects on bacterial virulence factors (ESAT-6), autophagy pathway (Beclin-1), macrophage polarization and apoptosis were evaluated in detail, aiming to reveal the potential mechanism of this pathway in regulating the immune microenvironment and maintaining immune escape in chronic MTB infection.

## 2 Materials and methods

### 2.1 Cell culture and transfection

RAW264.7 mouse macrophages were obtained from the Cell Bank of the Chinese Academy of Sciences (Shanghai) and maintained in DMEM supplemented with 10% FBS, 100 U/mL penicillin, and 100 µg/mL streptomycin at 37 °C under 5% CO_2_. For MCP-1 gene knockdown, cells were transfected with si*MCP-1* or a negative control siRNA (si-NC) using Lipofectamine™ RNAiMAX (Invitrogen, USA), following the manufacturer’s protocol.

### 2.2 Animals

Thirty-two male C57BL/6 mice (6–8 weeks old) were obtained from Anhui Ketu Biotechnology Co., Ltd. (Hefei, China) and housed under specific pathogen-free (SPF) conditions. Animal procedures were performed following the Guide for the Care and Use of Laboratory Animals (8th ed., 2011, NIH, USA) and conducted by Anhui Ketu Biotechnology. All protocols received ethical approval from the Animal Care and Use Committee of the First Affiliated Hospital of Xinxiang Medical College (approval no. dw-qn-2025-11).

### 2.3 H37Ra infection

The National Institutes for Food and Drug Control (Beijing, China) provided the attenuated MTB strain H37Ra. For *in vivo* experiments, mice were intranasally inoculated with a suspension of H37Ra (5 × 10^6^ CFU in 50 μL PBS) to establish a chronic pulmonary infection model, and samples were collected at 3 or 28 days post-infection. For *in vitro* stimulation, RAW264.7 macrophages were cultured in antibiotic-free medium prior to infection. Cells were then exposed to H37Ra at a multiplicity of infection (MOI) of 10 and cultured for various durations (8 or 48 hours), depending on the experimental setup. The bacterial suspension was pre-treated with polymyxin B to eliminate endotoxin contamination before use.

### 2.4 CCR2 antagonist administration

The CCR2-specific antagonist RS504393 (Selleck Chemicals, USA) was used to pharmacologically inhibit CCR2 signaling. For animal studies, mice received daily intraperitoneal injections of RS504393 at a dose of 2 mg/kg from day 21 to day 28 post-H37Ra infection.

### 2.5 Immunohistochemistry

Formalin-fixed (FFPE) lung tissues were sectioned at 4 μm. After deparaffinization/rehydration and antigen retrieval (15 min boiling in 10 mM citrate buffer, pH 6.0), endogenous peroxidase was blocked (3% H_2_O_2_, 10 min, RT). Sections were blocked with 5% BSA (30 min), then incubated overnight at 4 °C with primary antibodies: ESAT-6 (1:100, ab45073) and Beclin-1 (1:100, ab210498). After HRP-conjugated secondary antibody incubation (1:2000, ab205718, 30 min, RT), DAB detection and hematoxylin counterstaining were performed. Slides were imaged using a bright-field microscope (Leica DM5000, 200×), with positive cells quantified using ImageJ (version 1.53, NIH).

### 2.6 Quantitative real-time PCR

Total RNA was isolated from lung tissues and cultured macrophages with TRIzol reagent (Invitrogen). RNA purity/concentration was assessed using a NanoDrop 2000 spectrophotometer (Thermo Fisher Scientific). cDNA synthesis from 1 µg RNA employed the PrimeScript RT Reagent Kit (Takara, Japan) per the manufacturer’s protocol. qPCR was performed using SYBR Green Master Mix (Applied Biosystems, USA) on a QuantStudio 6 Flex Real-Time PCR System (Applied Biosystems). The relative mRNA expression levels were calculated using the 2^-ΔΔCt^ method, with *GAPDH* serving as the internal control. The primer sequences used for qPCR are listed in **Table 1**.

**Table 1:** qPCR primer sequences.

Gene	Forward primer (5’→3’)	Reverse primer (5’→3’)
*ESAT-6*	CGCAATTCGCCACCATGACAGAGCAGCAGTGGC	GCAGCCCCCAACGACCTTCTATGGCAACATCCCAGT
*Beclin-1*	GAGGAAGCTCAGTACCAGCG	CCAGATGTGGAAGGTGGCAT
*Trim30a*	TCTTGCCTGTGTTTGCTCCT	GTGTCTGCCTGTCCTGACTC
*Fas*	GTCAACCATGCCAACCTGAA	GCATCCACCCAAATCACCCC
*PD-1*	AGGTCCCTCACCTTCTACCC	GAAGGCGGCCTGTTTTTCAG
*MCP-1*	TGCCCTAAGGTCTTCAGCAC	AAGGCATCACAGTCCGAGTC
*Gapdh*	CCCTTAAGAGGGATGCTGCC	ATGAAGGGGTCGTTGATGGC

qPCR, Quantitative real-time PCR; *ESAT-6*, Early Secreted Antigenic Target 6; *Beclin-1*, Beclin-1; *TRIM30A*, Tripartite motif-containing 30A; *FAS*, Fas cell surface death receptor; *PD-1*, Programmed cell death protein 1; *MCP-1*, C-C motif chemokine ligand 2; *GAPDH*, Glyceraldehyde-3-phosphate dehydrogenase.

### 2.7 Hematoxylin and eosin staining

Harvested lung tissues underwent 24 h fixation in 10% neutral-buffered formalin, paraffin embedding, and 4 μm sectioning (Leica DM5000). Deparaffinized and rehydrated sections were H&E-stained: hematoxylin (5 min), eosin (2 min). Slides were dehydrated, mounted with neutral resin, and observed under a light microscope. Histopathological evaluation was performed to assess inflammatory cell infiltration and structural damage in the lung tissue.

### 2.8 Enzyme-linked immunosorbent assay

ELISA kits (Beyotime) measured mouse TNF-α (#PT512), IFN-γ (#PI508), TGF-β1 (#PT878), and IL-10 (#PI522) levels in mouse serum and macrophage supernatants. A Thermo Fisher microplate reader detected absorbance (450 nm), and cytokine levels were calculated based on the standard curves provided with each kit.

### 2.9 Western blot assay

Proteins were extracted from lung tissue and macrophages using RIPA buffer (Beyotime; protease inhibitors added). After BCA quantification (Beyotime kit), equivalent protein was resolved by SDS-PAGE and transferred to PVDF membranes (Millipore). Blocking was performed in 5% non-fat milk/TBST (1 h, RT). Membranes were then probed overnight at 4 °C with primary antibodies against TRIM30 (1:1000 dilution, ab76953, Abcam), PD-1 (1:1000 dilution, ab214421, Abcam), MCP-1 (1:1000 dilution, ab315478, Abcam), CD206 (1:1000 dilution, ab64693, Abcam), CD163 (1:1000 dilution, ab18242, Abcam), ARG1 (1:5000 dilution, Cat No. 16001-1-AP, Proteintech), CD86 (1:2000 dilution, Cat No. 83213-5-RR, Proteintech), Beclin-1 (1:1000 dilution, ab210498, Abcam), ESAT-6 (1:2000 dilution, ab45073, Abcam), and GAPDH (1:5000 dilution, ab8245, Abcam), followed by HRP-conjugated secondary antibodies (goat anti-rabbit or goat anti-mouse IgG, Abcam). An enhanced chemiluminescence (ECL) detection kit (Thermo Fisher Scientific, USA) was used to view the bands, and ImageJ software (NIH, version 1.53) was used to quantify them.

### 2.10 Macrophage polarization analysis

To assess macrophage polarization, flow cytometry and immunofluorescence staining were performed to evaluate the expression of M2 surface markers CD163 and CD206. Briefly, cells were harvested and incubated with anti-F4/80 (BioLegend, Cat#123110, USA), anti-CD206-PE (BioLegend, Cat#141706, USA), and anti-CD163-APC (BioLegend, Cat#155310, USA) antibodies for 30 minutes at 4 °C in the dark. The samples were examined using a BD FACSCanto™ II flow cytometer (BD Biosciences, USA) following two PBS washes. Software called FlowJo (FlowJo, LLC) was used to process the data.

For immunofluorescence, macrophages on coverslips were fixed with 4% paraformaldehyde for 15 minutes, permeabilized with 0.1% Triton X-100, and blocked with 5% BSA for 1 hour (RT). Cells were incubated overnight at 4 °C with primary antibodies against CD206 (Abcam, ab64693) or CD163 (Abcam, ab182422), followed by incubation with Alexa Fluor^®^ 594/488 secondary antibodies (Thermo Fisher Scientific) for 1 hour at RT. Nuclei were stained with DAPI. Images were captured using a Nikon Eclipse Ti fluorescence microscope and analyzed with ImageJ software (NIH, version 1.53).

### 2.11 Flow cytometric analysis of apoptosis

Apoptosis of macrophages was evaluated using Annexin V-FITC/propidium iodide (PI) double staining. Cells were harvested at the indicated time points and washed twice with cold PBS, followed by resuspension in 100 μL of 1× binding buffer. For 15 minutes at room temperature in the dark, cell suspensions were stained with 5 μL of Annexin V-FITC and 5 μL of PI (BD Biosciences Annexin V-FITC/PI Apoptosis Detection Kit). 400 μL of 1× binding buffer was added after incubation, and samples were examined right away using a BD FACSCanto II flow cytometer (BD Biosciences). To analyze the data, FlowJo software (FlowJo LLC) was used.

### 2.12 RNA sequencing and bioinformatics analysis

TRIzol reagent (Invitrogen) was used to extract total RNA from mouse lung tissue. Thermo Scientific’s NanoDrop 2000 and Agilent Technologies’ Agilent 2100 Bioanalyzer were used to confirm the integrity of the RNA. Shanghai Majorbio Bio-pharm Technology was responsible for the Illumina NovaSeq 6000 sequencing. Clean reads were aligned to the GRCm39 mouse genome using HISAT2 after adapter and quality trimming, and then StringTie was used to quantify gene expression. DESeq2 applied criteria of |log_2_Fold Change| > 1 and adjusted *P* < 0.05 to identify differentially expressed genes (DEGs). GO and KEGG enrichment analyses were performed using the clusterProfiler R package to investigate biological processes and pathways associated with CCR2 signaling.

### 2.13 Statistical analysis

The mean ± standard deviation (SD) is used to display all data. Unpaired Student’s t-test or one-way ANOVA followed by Tukey’s *post hoc* test, if applicable, were used to assess statistical significance. Statistical significance was defined as a *P*-value of less than 0.05. GraphPad Prism 9.0 (GraphPad Software, USA) and R software (version 4.3.1) were used to create graphs and conduct statistical analyses for data visualization and analysis relevant to bioinformatics.

## 3 Results

### 3.1 CCR2 inhibition downregulates ESAT-6 expression and increases *Beclin-1* levels in lung tissues of H37Ra-infected mice

**Figure 1:**
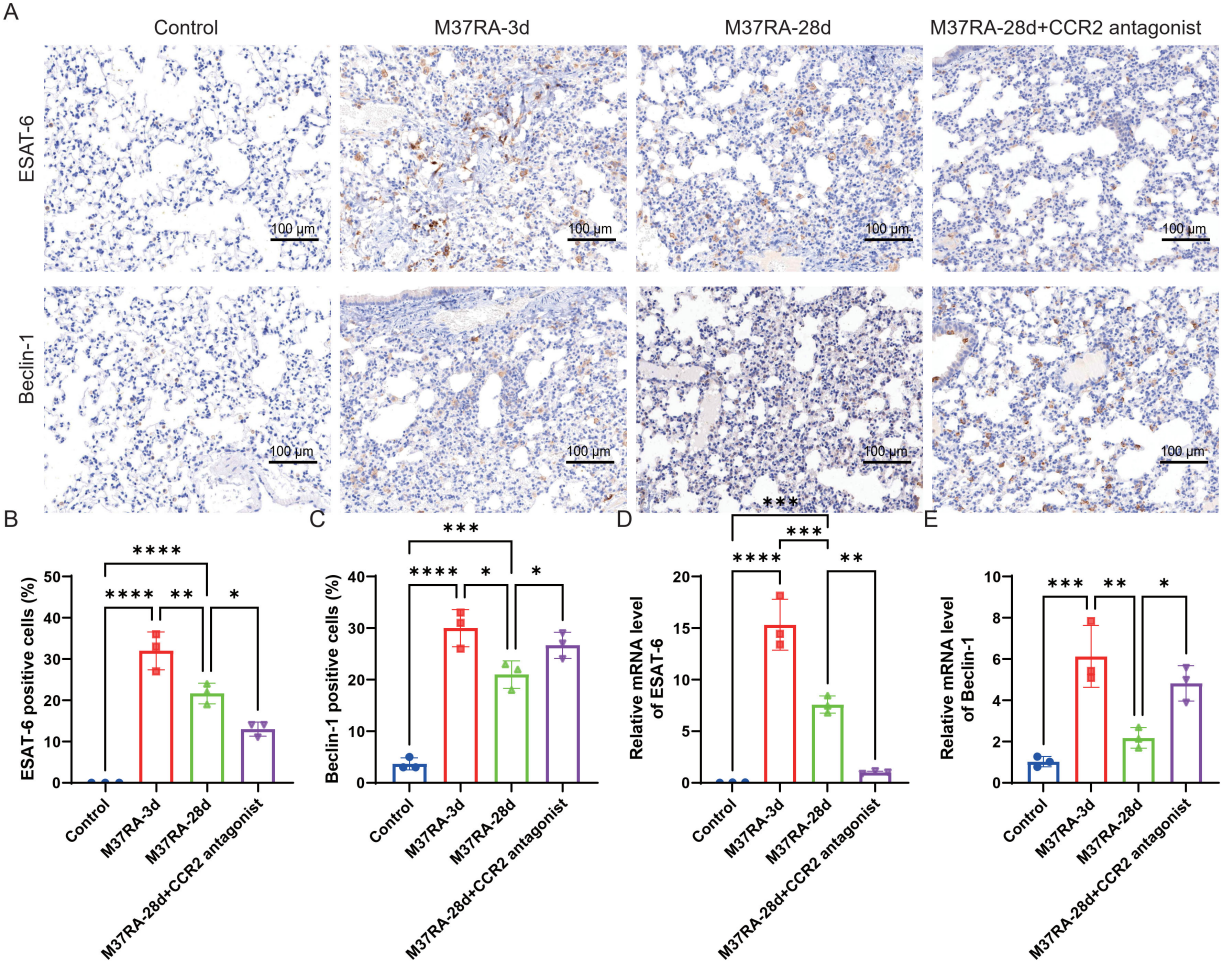
Expression levels of the virulence marker ESAT-6 and the autophagy marker Beclin-1 in lung tissues of H37Ra-infected mice. **(A)** Representative IHC staining of ESAT-6 and Beclin-1 in lung sections of mice in the control, H37Ra-3d, H37Ra-28d, and H37Ra-28d+*CCR2* antagonist groups. Scale bar = 100 μm. **(B, C)** Quantitative analysis of ESAT-6-positive **(B)** and Beclin-1-positive **(C)** cells based on IHC analysis. **(D, E)** qPCR analysis of *ESAT-6***(D)** and *Beclin-1***(E)** mRNA levels in lung sections of mice in the control, H37Ra-3d, H37Ra-28d, and H37Ra-28d+*CCR2* antagonist groups. *P < 0.05, **P < 0.01, ***P < 0.001, ****P < 0.0001. IHC, Immunohistochemistry; qPCR, Quantitative Polymerase Chain Reaction; d, day; *CCR2*, C-C motif chemokine receptor 2; ESAT-6, Early Secreted Antigenic Target 6; Beclin-1, Beclin-1.

Studies have shown that *CCR2* plays a key role in MTB infection, mainly involving monocyte recruitment, immune regulation, and disease pathogenesis (14, 15). In this study, we evaluated the expression of MTB virulence marker ESAT-6 and autophagy marker Beclin-1 in the lung tissues of mice infected with H37Ra. IHC staining indicated that the percentage of ESAT-6-positive cells increased significantly by day 3 after infection and gradually decreased by day 28. Notably, administering *CCR2* antagonists on day 28 further reduced ESAT-6 expression compared with mice infected with H37Ra for 28 days. In contrast, Beclin-1 expression was significantly upregulated on day 3 but decreased by day 28 after CCR2 antagonist, with partial recovery following the administration of the *CCR2* antagonist (**Figure 1A, C**). qPCR analysis consistently confirmed that *ESAT-6* mRNA levels peaked on day 3 and declined by day 28, while *Beclin-1* mRNA levels showed a similar pattern (**Figure 1D, E**). Notably, *CCR2* antagonist treatment resulted in a significant decrease in *ESAT-6* mRNA levels and an increase in *Beclin-1* mRNA levels compared to the H37Ra-28d control group. These results suggest that CCR2 signaling may modulate the host environment in a manner that influences mycobacterial virulence markers.

### 3.2 CCR2 inhibition restores lung inflammation and reduces lung tissue damage in H37Ra-infected mice

**Figure 2:**
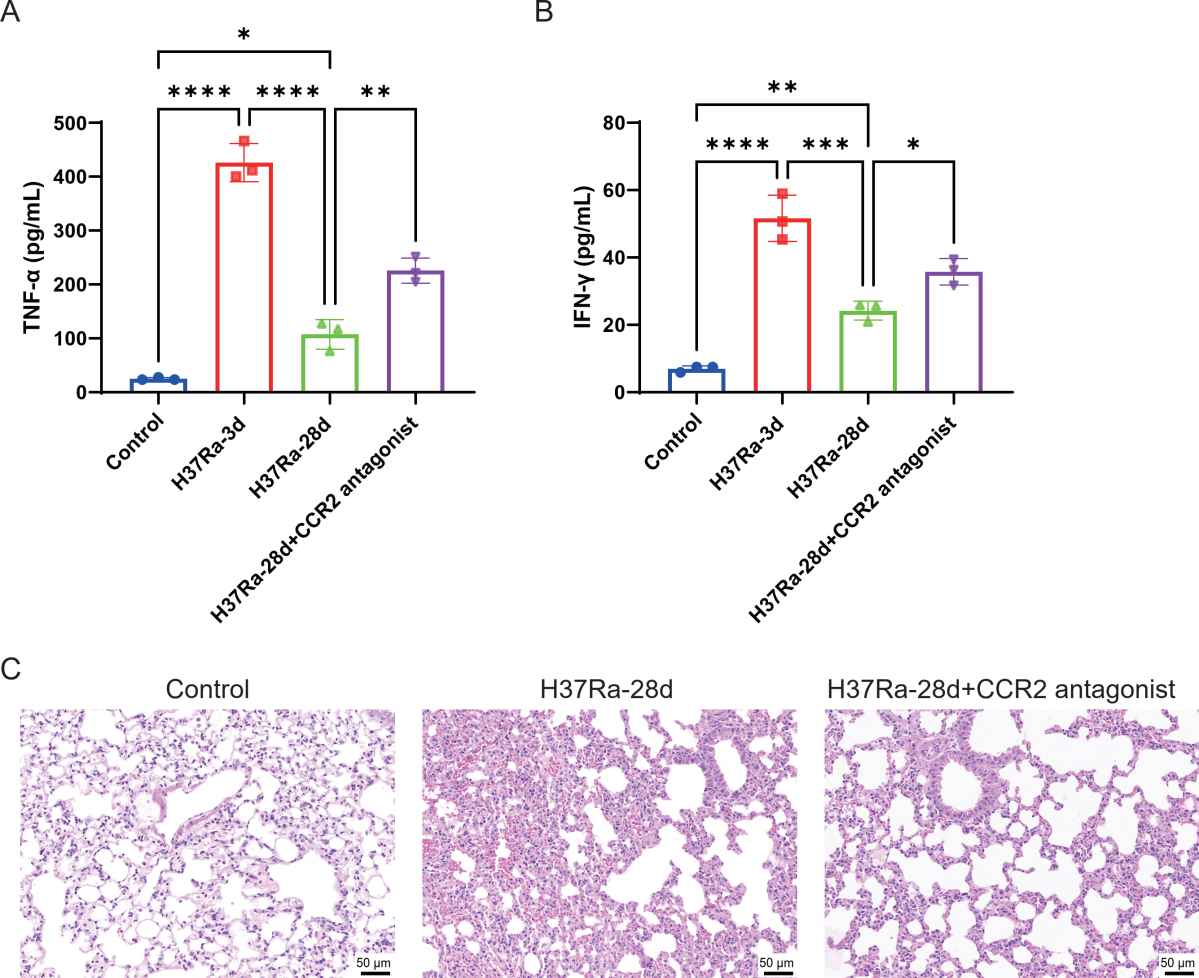
*CCR2* inhibition restores proinflammatory cytokine levels and reduces lung tissue damage in H37Ra-infected mice. **(A, B)** ELISA quantification of serum TNF-α **(A)** and IFN-γ **(B)** in lung homogenates of the control, H37Ra-3d, H37Ra-28d, and H37Ra-28d+*CCR2* antagonist groups. **(C)** Representative lung tissue sections stained with H&E to show histopathological changes. Scale bar = 50 μm. *P < 0.05, **P < 0.01, ***P < 0.001, ****P < 0.0001. ELISA, Enzyme-Linked Immunosorbent Assay; TNF-α, Tumor Necrosis Factor-alpha; IFN-γ, Interferon-gamma; H&E, Hematoxylin and Eosin; d, day; *CCR2*, C-C motif chemokine receptor 2.

To explore the effects of CCR2 signaling on inflammatory responses and lung pathology in mice infected with H37Ra, we quantified serum TNF-α and IFN-γ and examined lung histopathology. As shown in **Figures 2A, B**, H37Ra infection significantly increased the production of serum TNF-α and IFN-γ on day 3, which gradually decreased on day 28. However, the addition of CCR2 antagonists on day 28 partially restored the levels of TNF-α and IFN-γ caused by H37Ra infection, suggesting that immunosuppression was reversed. Histological analysis of H&E staining showed severe inflammatory cell infiltration and alveolar structural destruction in the H37Ra-28d group (**Figure 2C**). However, *CCR2* blockade significantly alleviated these pathological changes. These results suggest that the *CCR2* antagonist may assist in restoring immune activity, alleviating excessive inflammation, and improving lung tissue lesions in the late stage of infection.

### 3.3 Transcriptomic profiling reveals CCR2-associated transcriptional alterations and pathway enrichment in H37Ra-induced pulmonary tuberculosis model

**Figure 3:**
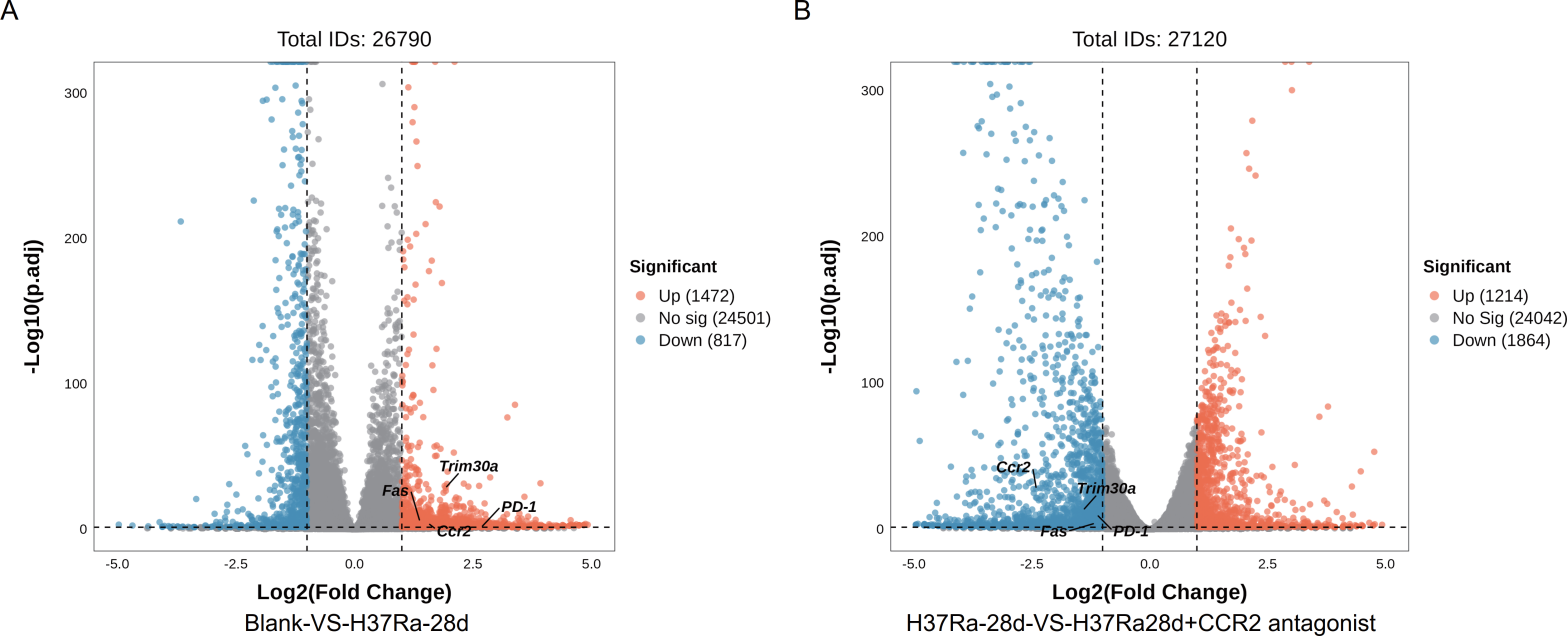
Transcriptomic alterations in lung tissues upon H37Ra infection and *CCR2* inhibition. **(A, B)** Volcano plots showing differentially expressed genes (DEGs) between Blank and H37Ra-28d groups **(A)**, and between H37Ra-28d and H37Ra-28d+*CCR2* antagonist groups **(B)**. Red and blue dots indicate significantly upregulated and downregulated genes, respectively. Genes of interest (*Trim30a*, *Fas*, *PD-1*, *Ccr2*) are labeled. DEGs, Differentially expressed genes; *Ccr2*, C-C motif chemokine receptor 2; d, day; *Trim30a*, Tripartite motif-containing 30A; *Fas*, Fas cell surface death receptor; *PD-1*, Programmed cell death protein 1.

**Figure 4:**
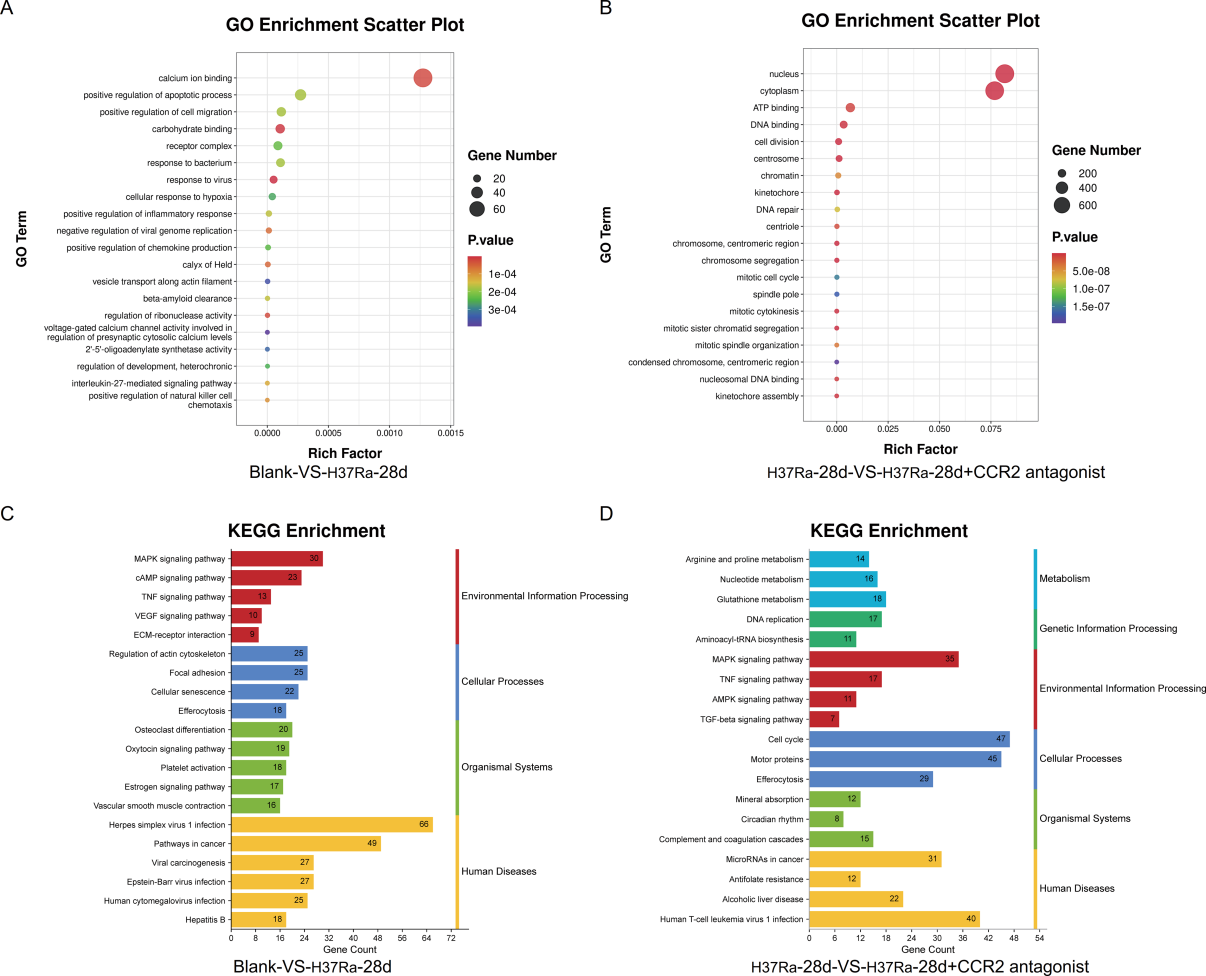
GO and KEGG enrichment analyses of DEGs. **(A, B)** GO enrichment scatter plots of DEGs between Blank *vs* H37Ra-28d **(A)** and H37Ra-28d *vs* H37Ra-28d+*CCR2* antagonist groups **(B)**. Each dot represents a GO term; dot size and color indicate gene count and adjusted *P*-value, respectively. **(C)** KEGG enrichment of DEGs between the Blank and H37Ra-28d groups. Enriched pathways are classified into four categories: Environmental Information Processing, Cellular Processes, Organismal Systems, and Human Diseases. **(D)** KEGG enrichment of DEGs between the H37Ra-28d and H37Ra-28d+*CCR2* antagonist groups. Enriched pathways are classified into six categories: Metabolism, Genetic Information Processing, Environmental Information Processing, Cellular Processes, Organismal Systems, and Human Diseases. GO, Gene Ontology; KEGG, Kyoto Encyclopedia of Genes and Genomes; DEGs, Differentially expressed genes; *CCR2*, C-C motif chemokine receptor 2; d, day.

To investigate transcriptomic changes associated with H37Ra-induced lung responses and the effects of *CCR2* antagonists, we performed RNA sequencing and differential gene expression analysis. The volcano plot showed that 1,472 genes were upregulated and 817 genes were downregulated in the blank and H37Ra-28d groups (**Figure 3A**); 1,214 genes were upregulated and 1,864 genes were downregulated in the H37Ra-28d and H37Ra-28d+*CCR2* antagonist groups (**Figure 3B**). Among them, *Trim30a*, *Fas*, *PD-1*, and *Ccr2* were significantly upregulated in H37Ra-28d lung tissues, but downregulated after *CCR2* antagonist, suggesting that they are *CCR2*-dependent. GO enrichment analysis indicated that DEGs in the H37Ra-28d group were significantly enriched in biological processes such as “positive regulation of apoptotic process,” “positive regulation of cell migration,” and “response to bacterium” (**Figure 4A**) In contrast, the *CCR2* antagonist group showed prominent enrichment in cell cycle-related terms such as “chromosome segregation” and “DNA repair” (**Figure 4D**). KEGG pathway analysis further supported these findings, highlighting activation of immune-inflammatory signaling in the H37Ra-28d group, such as TNF, MAPK, and VEGF signaling pathways (**Figure 4C**). However, *CCR2* antagonist resulted in enrichment of cell cycle-related and metabolic pathways, including “DNA replication,” “glutathione metabolism,” and “cell cycle,” indicating a shift in cellular processes from inflammation to proliferation (**Figure 4D**).

### 3.4 MCP-1/CCR2 axis mediates apoptosis of macrophages stimulated by H37Ra

**Figure 5:**
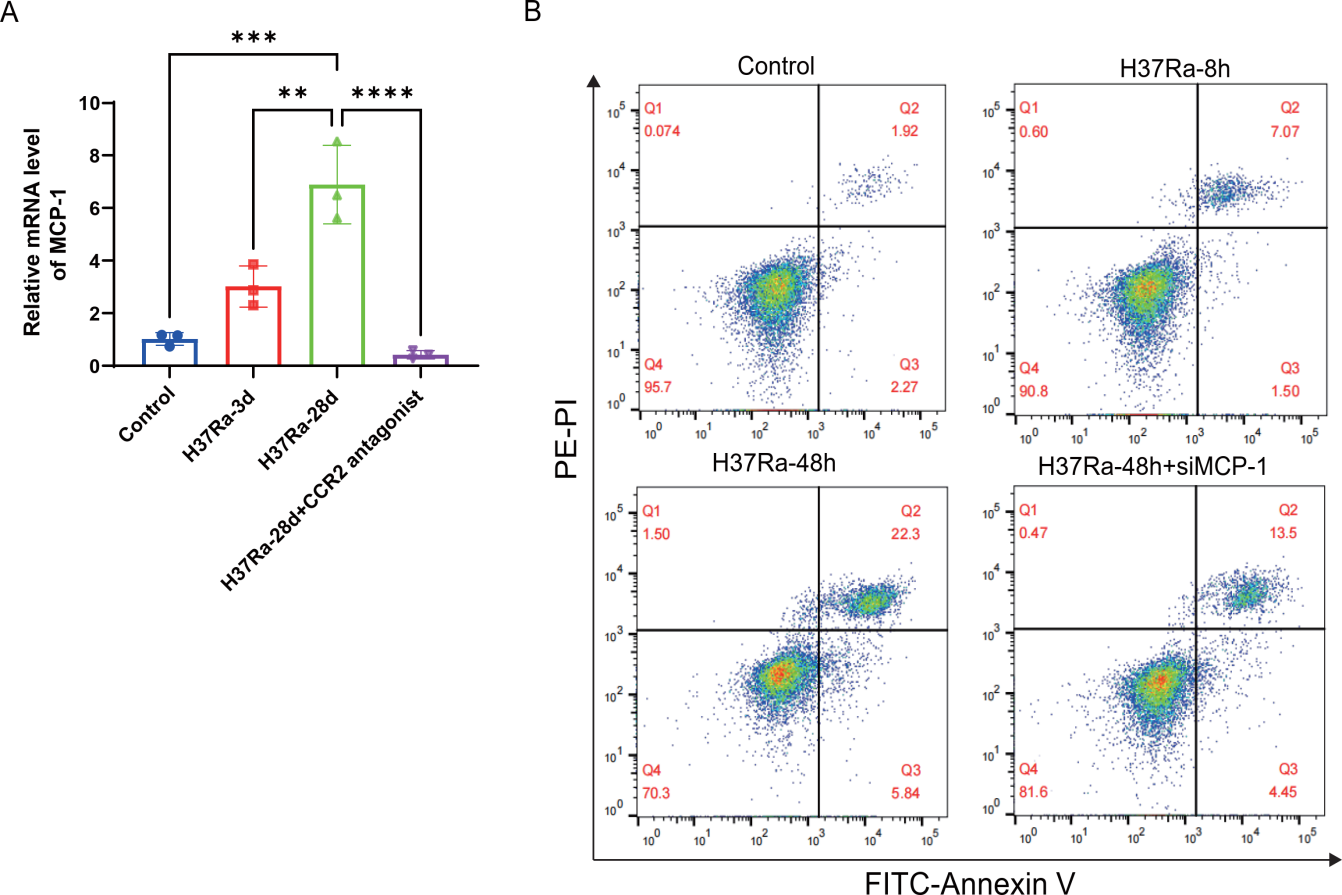
*MCP-1* expression and apoptosis analysis of RAW264.7 macrophages after H37Ra stimulation. **(A)** qPCR detection of *MCP-1* mRNA levels at days 3, 28, and 28 days plus CCR2 antagonist after H37Ra infection *in vivo*. **(B)** Flow cytometry analysis of macrophage apoptosis using Annexin V-FITC and PE-PI staining after H37Ra stimulation for 8 h or 48 h, or 48 h with *MCP-1* siRNA transfection. Q3 (Annexin V^+^/PI^-^) indicates early apoptosis; Q2 (Annexin V^+^/PI^+^) indicates late apoptosis. **P < 0.01, ***P < 0.001, ****P < 0.0001. *CCR2*, C-C motif chemokine receptor 2; *MCP-1*, C-C motif chemokine ligand 2.

To explore the regulatory role of the *MCP-1*/*Ccr2* axis in H37Ra-induced responses, we examined *in vivo MCP-1* expression in the mouse model. qPCR of mouse lungs showed that H37Ra infection increased *MCP-1* mRNA at 3 days and peaked at 28 days, whereas treatment with the CCR2 antagonist RS504393 markedly reduced *MCP-1* levels (**Figure 5A**). To further elucidate the functional impact of *MCP-1*, we performed Annexin V/PI staining to evaluate the apoptosis level of macrophages after H37Ra exposure. As shown in **Figure 5B**, the number of early (Q3) and late (Q2) apoptotic cells increased in a time-dependent manner after 8 and 48 hours of H37Ra stimulation. Notably, knockdown of the MCP-1 gene using siRNA reduced apoptosis after 48 hours of stimulation, attenuating the number of early apoptotic cells from 22.3% to 13.5%. These results suggest that *MCP-1* knockdown may play a protective anti-apoptotic role in macrophages through *Ccr2*-mediated signaling.

### 3.5 MCP-1 promotes H37Ra-induced M2 macrophage polarization

**Figure 6:**
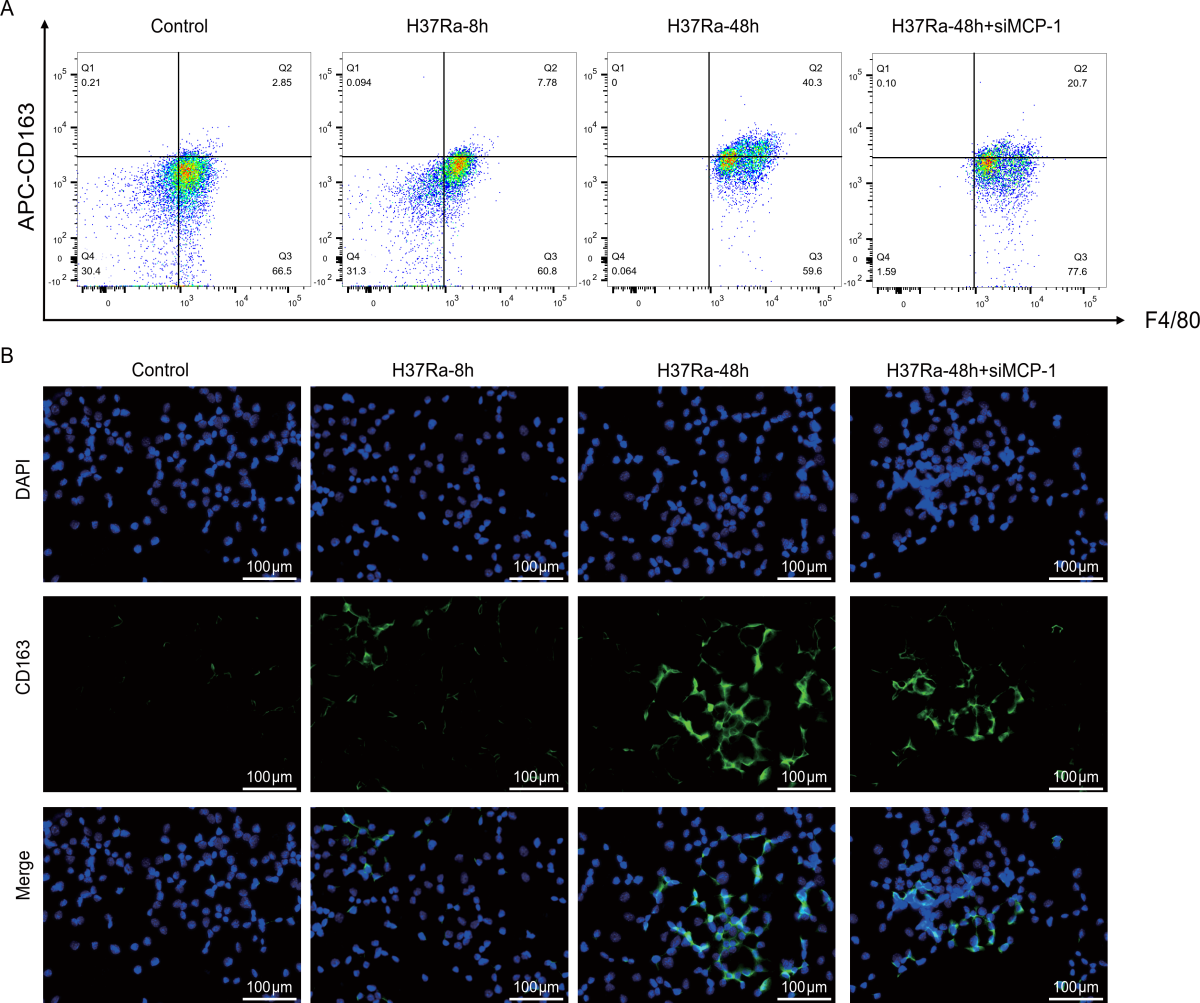
Flow cytometry and immunofluorescence analysis of CD163 expression in macrophages. **(A)** Representative flow cytometry plots showing the proportion of CD163^+^F4/80^+^ cells in control, H37Ra-8h, H37Ra-48h, and H37Ra-48h+si*MCP-1* groups. **(B)** Immunofluorescence staining for CD163 (green) and DAPI (blue) in macrophages under the indicated treatments. Scale bar = 100 μm. *MCP-1*, C-C motif chemokine ligand 2; CD163, Cluster of Differentiation 163.

**Figure 7:**
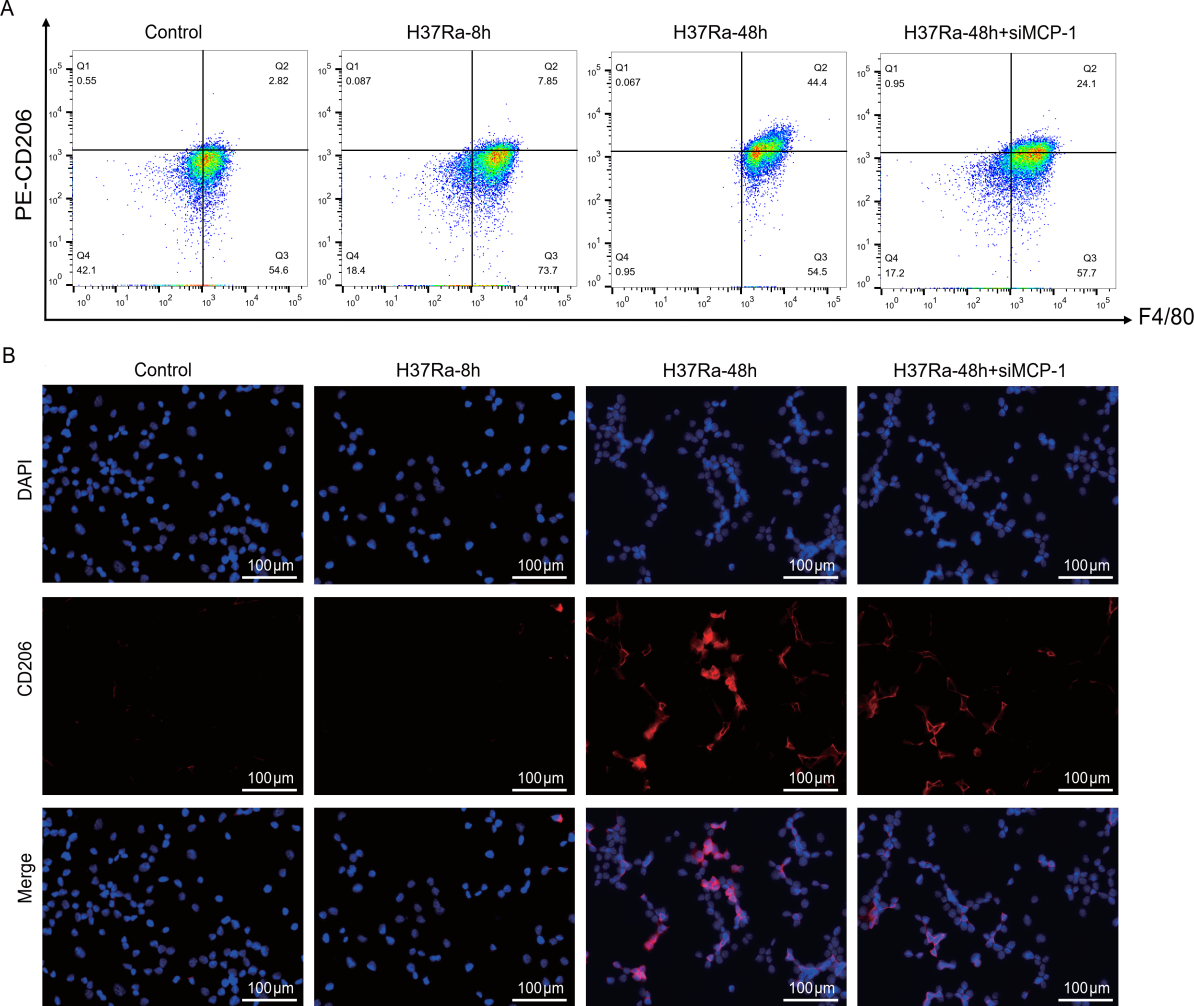
Flow cytometry and immunofluorescence analysis of CD206 expression in macrophages. **(A)** Flow cytometry plots showing the percentage of CD206^+^F4/80^+^ cells in control, H37Ra-8h, H37Ra-48h, and H37Ra-48h+si*MCP-1* groups. **(B)** Immunofluorescence images of CD206 (red) and DAPI (blue) staining in macrophages across different treatment conditions. Scale bar = 100 μm. *MCP-1*, C-C motif chemokine ligand 2; CD206, Mannose receptor C-type 1.

Studies have found that MTB can promote its survival and chronic infection by inducing macrophages to polarize toward M2 type and inhibiting their bactericidal function (16, 17). To explore the effect of *MCP-1* on H37Ra-induced macrophage polarization, we first evaluated the expression of the M2 marker CD163. Flow cytometry analysis showed that after H37Ra stimulation, CD163^+^F4/80^+^ macrophages gradually increased, especially at 48 hours, while siRNA-mediated *MCP-1* knockdown significantly reduced the proportion of CD163^+^ cells (**Figure 6A**). Immunofluorescence staining results consistently confirmed that H37Ra stimulation enhanced the expression of CD163, while *MCP-1* knockdown significantly inhibited the signal intensity of CD163 (**Figure 6B**). Similarly, analysis of another M2 marker, CD206, showed a similar trend. H37Ra stimulation induced a time-dependent increase in the number of CD206 macrophages, while *MCP-1* knockdown strongly inhibited this increase (**Figure 7A**). Immunofluorescence staining further verified the increase in CD206 expression after H37Ra stimulation and the decrease in CD206 expression after *MCP-1* knockdown (**Figure 7B**). These findings suggest that *MCP-1* is involved in H37Ra-induced M2 macrophage polarization.

### 3.6 MCP-1 regulates the levels of inflammatory cytokines and ESAT-6/autophagy-related proteins in macrophages

**Figure 8:**
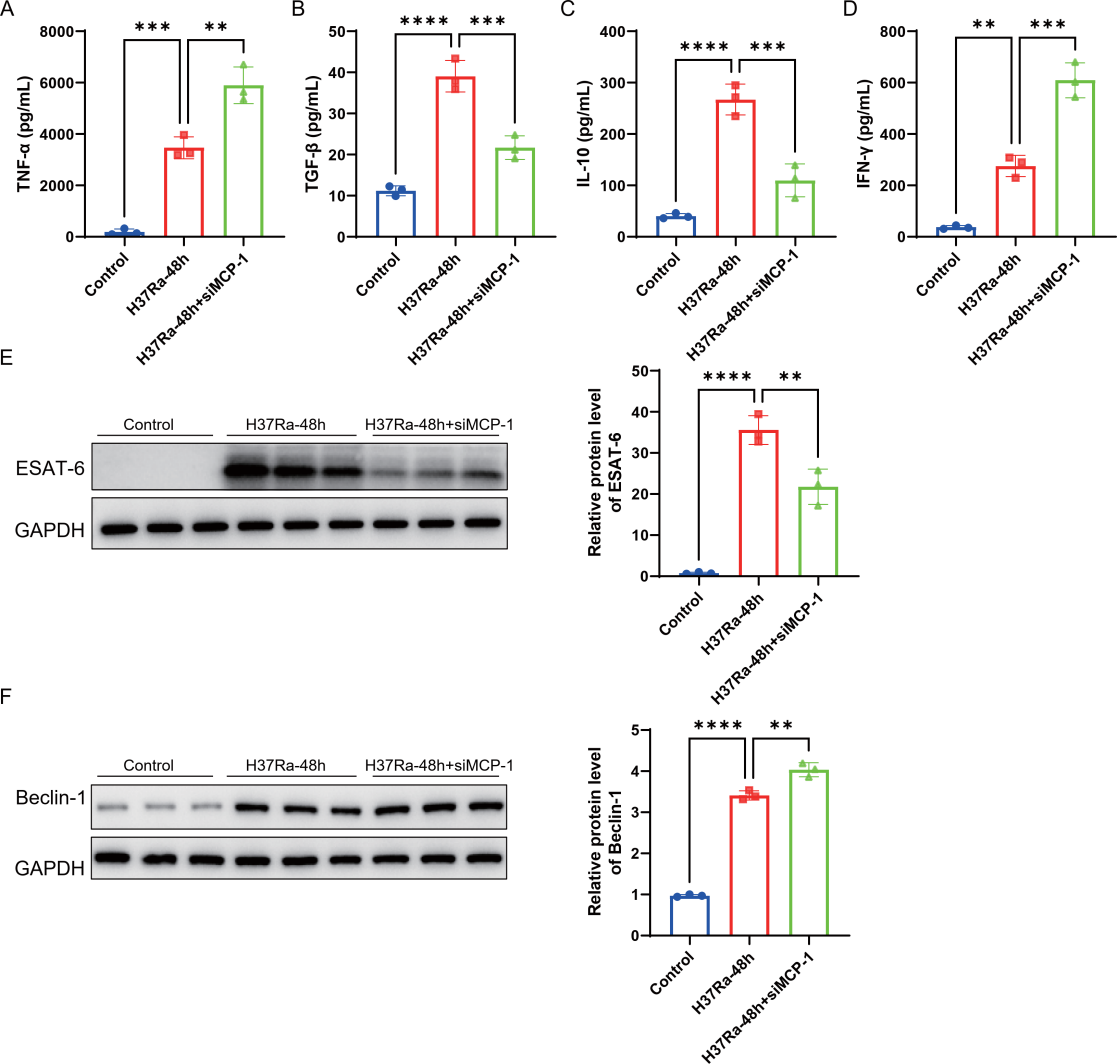
*MCP-1* regulates the levels of inflammatory cytokines and ESAT-6/autophagy-related proteins in macrophages. **(A-D)** ELISA analysis of TNF-α **(A)**, TGF-β **(B)**, IL-10 **(C)**, and IFN-γ **(F)** in the supernatants of RAW264.7 macrophages treated with control, H37Ra-48h, or H37Ra-48h+si*MCP-1*. **(E-F)** Western blot and densitometry analysis of ESAT-6 **(E)** and Beclin-1 **(F)** protein levels in different treatment groups; GAPDH was used as a loading control. **P < 0.01, ***P < 0.001, ****P < 0.0001. *MCP-1*, C-C motif chemokine ligand 2; ELISA, Enzyme-Linked Immunosorbent Assay; MTB, Mycobacterium tuberculosis; TNF-α, Tumor Necrosis Factor-alpha; TGF-β, Transforming Growth Factor-beta; IL-10, Interleukin-10; IFN-γ, Interferon-gamma; ESAT-6, Early Secreted Antigenic Target 6; Beclin-1, Beclin-1.

We then analyzed the role of *MCP-1* in regulating the expression of inflammatory cytokines and ESAT-6/autophagy-related markers. In ELISA experiments, H37Ra stimulation for 48h significantly upregulated the levels of TNF-α, TGF-β, IL-10, and IFN-γ in the supernatant of RAW264.7 macrophages compared with the control group (**Figures 8A, D**). Knockdown of *MCP-1* further enhanced the production of TNF-α and IFN-γ, while significantly reducing the levels of TGF-β and IL-10. Western blot analysis showed that H37Ra treatment for 48h significantly increased the protein levels of ESAT-6 and Beclin-1 (**Figures 8E, F**). Knockdown of *MCP-1* significantly reduced the expression of ESAT-6, but further promoted the upregulation of Beclin-1. These results suggest that *MCP-1* may regulate the secretion of inflammatory cytokines and play opposite roles in regulating the virulence factor ESAT-6 and the autophagy-related protein Beclin-1 in H37Ra-stimulated macrophages.

### 3.7 MCP-1 promotes M2 polarization and immunosuppressive signaling in H37Ra-treated macrophages

**Figure 9:**
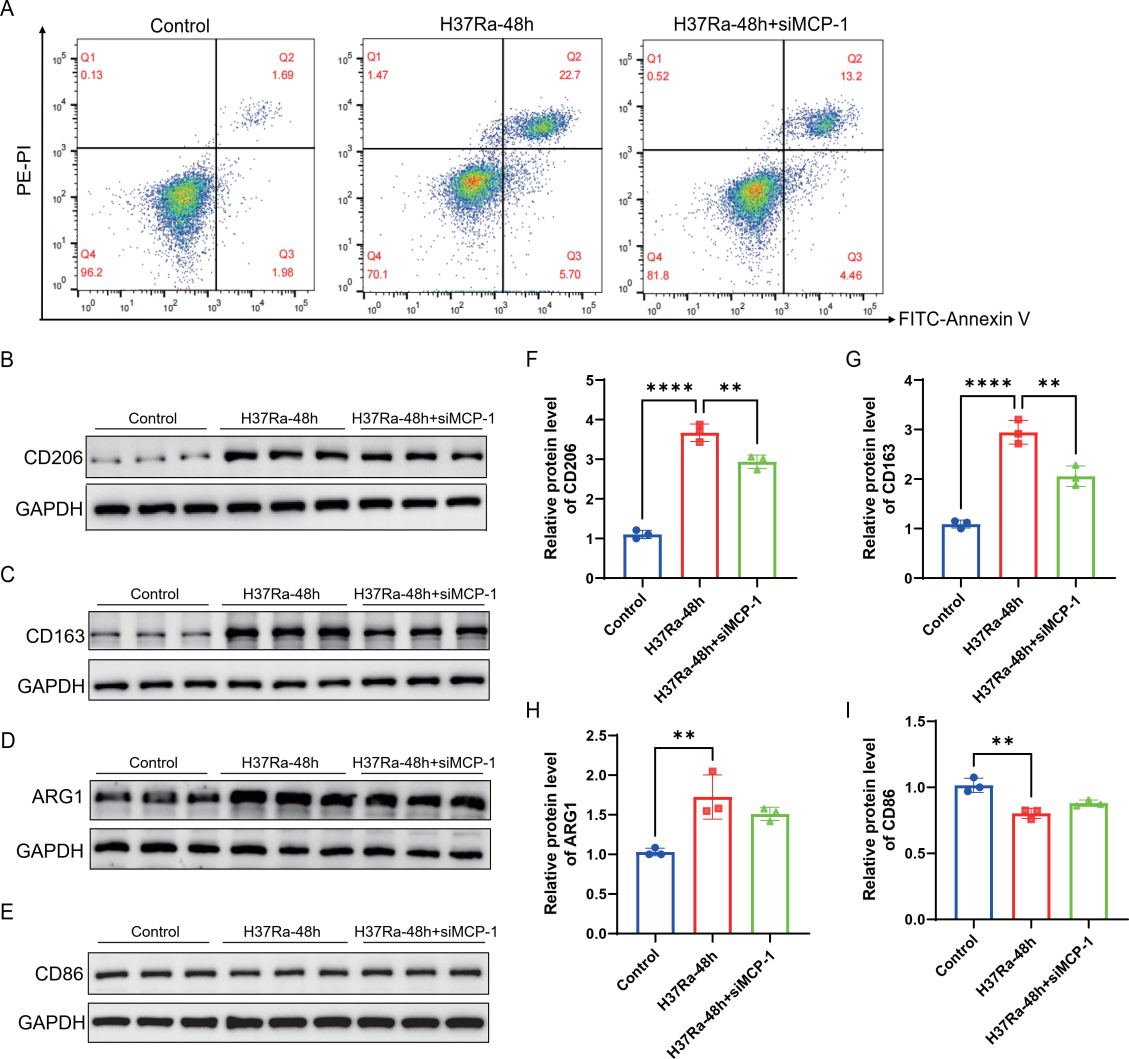
*MCP-1* knockdown modulates M1/M2 macrophage polarization markers and apoptosis. **(A)** Apoptosis of macrophages under control, H37Ra-48h, and H37Ra-48h+si*MCP-1* conditions was analyzed by flow cytometry using Annexin V/PI staining. **(B-E)** Representative Western blot images showing protein expression of CD206 **(B)**, CD163 **(C)**, ARG1**(D)** (M2 marker), and CD86 **(E)** (M1 marker). **(F-I)** Quantitative analysis of CD206 **(F)**, CD163 **(G)**, ARG1 **(H)**, and CD86 **(I)** protein levels (normalized to GAPDH). **P < 0.01, ****P < 0.0001. *MCP-1*, C-C motif chemokine ligand 2; CD206, C-type lectin domain family 13 member D; CD163, Cluster of Differentiation 163; ARG1, Arginase 1; CD86, Cluster of Differentiation 86.

**Figure 10:**
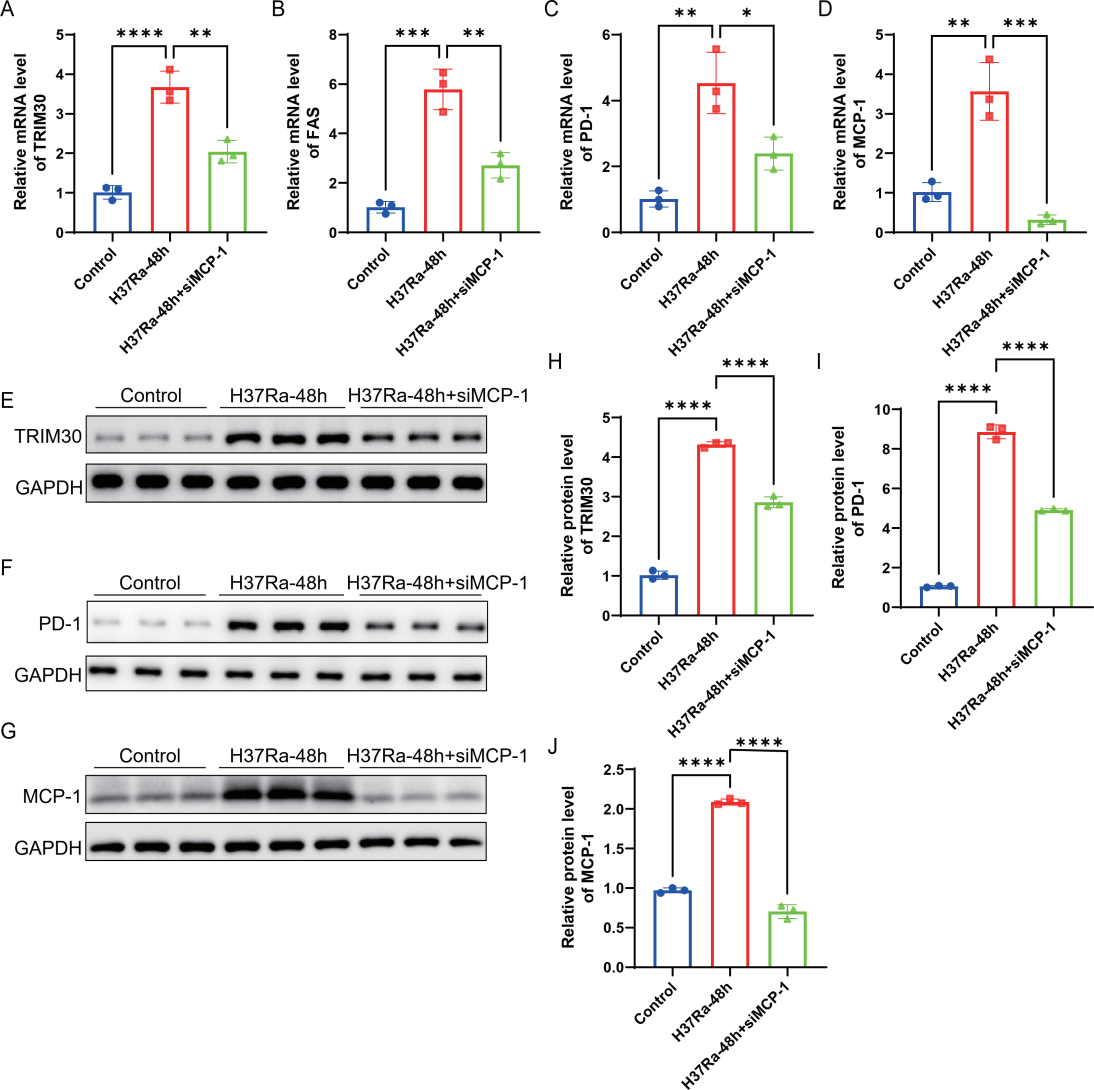
*MCP-1* regulates immunosuppressive gene expression in macrophages. **(A-D)** Quantitative real-time PCR analysis of mRNA levels of *Trim30***(A)**, *Fas***(B)**, *PD-1***(C)**, and *MCP-1****(*D*)*** in control, H37Ra-48h, and H37Ra-48h+si*MCP-1* macrophages. **(E-G)** Western blot analysis of TRIM30 **(E)**, PD-1 **(F)**, and MCP-1 **(G)** protein expression, with GAPDH as a loading control. **(H-J)** Quantification of protein band intensities corresponding to TRIM30 **(H)**, PD-1 **(I)**, and MCP-1 **(J)**, respectively. *P < 0.05, **P < 0.01, ***P < 0.001, ****P < 0.0001. *MCP-1*, C-C motif chemokine ligand 2; *Trim30*, Tripartite motif-containing 30A; *Fas*, Fas cell surface death receptor; *PD-1*, Programmed cell death protein 1.

Further experiments investigated the role of *MCP-1* in regulating macrophage polarization and immunosuppressive markers. Flow cytometry results showed that compared with the control group, H37Ra treatment for 48h significantly increased the number of early and late apoptotic macrophages, while *MCP-1* knockdown reduced the proportion of apoptotic cells (**Figure 9A**). Western blot analysis further showed that H37Ra treatment for 48h upregulated the expression of M2 markers CD206, CD163, and ARG1, while the expression of CD206 and CD163 decreased significantly after *MCP-1* knockdown, but the inhibitory effect of ARG1 was relatively weak (**Figures 9B, C, D, F, G, H**). In contrast, the expression of M1 marker CD86 was downregulated after H37Ra treatment for 48h and slightly restored after *MCP-1* knockdown, although the difference was not significant (**Figures 9E, I**). qPCR analysis results showed that H37Ra stimulation for 48h significantly increased the mRNA levels of *Trim30*, *Fas*, *PD-1*, and *MCP-1*, while *MCP-1* knockdown significantly reduced the expression levels of these molecules (**Figures 10A-D**). Western blot further confirmed the protein expression patterns of TRIM30, PD-1, as well as MCP-1, which were consistent with the transcriptome data (**Figures 10E-J**). These data suggest that *MCP-1* may be involved in H37Ra-induced M2 polarization, anti-inflammatory state, and immunosuppressive signaling.

## 4 Discussion

Using a mouse model of pulmonary tuberculosis caused by the attenuated strain H37Ra, we methodically examined the function of the *MCP-1/Ccr2* signaling axis in immunoregulation during MTB infection. Our findings imply that, via modifying mycobacterial pathogenicity, autophagy, inflammatory responses, and macrophage function, *Ccr2* signaling is essential for controlling host-pathogen interactions. Specifically, inhibition of *CCR2* significantly reduced the expression of the MTB virulence factor ESAT-6, while restoring the level of the autophagy marker Beclin-1 and attenuating lung tissue damage. Transcriptome analysis revealed a *Ccr2*-dependent immunosuppressive gene signature, including *TRIM30A*, *PD-1*, and *FAS*, which was reversed after *CCR2* blockade. Furthermore, *MCP-1* mediated macrophage anti-apoptosis and promoted M2 polarization, while upregulating immunosuppressive markers such as CD206, CD163, and ARG1. Together, these results suggest that *MCP-1/Ccr2* signaling contributes to persistent infection by orchestrating a suppressive immune microenvironment and modulating the host environment in a manner that influences mycobacterial virulence markers.

ESAT-6 is a key virulence factor of MTB, which can promote bacterial escape to the cytoplasm by disrupting the host cell membrane. It directly binds to and inhibits the autophagic protein, blocking autophagosome formation, thereby helping bacteria evade host autophagic clearance (18). At the same time, inhibition of Beclin-1 function disrupts cellular homeostasis and exacerbates inflammatory damage at the site of infection (19). This interference mechanism targeting autophagy is an important strategy for MTB to achieve immune escape and intracellular survival. In the lungs of mice infected with H37Ra, we found that suppressing *CCR2* significantly decreased ESAT-6 expression while restoring Beclin-1 levels. These findings suggest that *MCP-1/Ccr2* signaling facilitates mycobacterial persistence by enhancing virulence (reflected by increased ESAT-6) and suppressing autophagy (via reduced Beclin-1). In parallel, *MCP-1/Ccr2* promotes macrophage polarization toward an M2 phenotype, which is generally associated with diminished bactericidal activity. TNF-α and IFN-γ are key inflammatory factors for the host to resist MTB infection (20), of which TNF-α regulates granuloma formation, bacterial dissemination, and tissue clearance (21), whereas IFN-γ boosts bactericidal activity by activating macrophages (22). Our study found that H37Ra infection decreased lung tissue’s production of TNF-α and IFN-γ after 28 days, while *CCR2* antagonists could significantly restore their expression levels, suggesting that *Ccr2* may be involved in regulating the establishment of an immunosuppressive state during chronic infection. This result further suggests that blocking *Ccr2* signals can help enhance the host’s immune response and reduce lung inflammatory damage.

Transcriptome analysis further confirmed the immunomodulatory role of *CCR2* signaling in chronic MTB infection. *CCR2* blockade reversed the upregulation of immunosuppressive and pro-apoptotic genes (*TRIM30*, *FAS*, *PD-1*, and *CCR2*), indicating that these genes are dependent on CCR2 signaling. Among them, *TRIM30*, as an E3 ubiquitin ligase, can negatively regulate the NLRP3 inflammasome pathway and inhibit excessive inflammatory responses (23); *FAS* participates in the apoptotic clearance of infected cells, which helps to limit the continued survival of pathogens in the host (24). Notably, *PD-1* is crucial for preserving immunological homeostasis and avoiding over-activation of the immune system (25, 26). The literature (27) reports that *PD-1*-deficient patients inactivate *PD-1* signals in leukocytes, resulting in reduced IFN-γ secretion and dysfunction of T cell subsets, thereby increasing susceptibility to MTB infection. In addition, the literature also pointed out that PD-1 dysregulation can trigger excessive activation of *STAT3*-related pathways, further promoting immune abnormalities and autoimmune pulmonary lesions. Our GO enrichment analysis showed that chronic infection was associated with activation of pathways related to inflammation, cell migration, and apoptosis, Our GO enrichment analysis revealed that persistent infection activated pathways linked to inflammation, cell migration, and death, whereas *CCR2* inhibition redirected gene expression toward cell cycle regulation as well as DNA repair. In addition, KEGG analysis showed that *CCR2* signaling promoted proinflammatory pathways (e.g., TNF, MAPK, VEGF), while *CCR2* inhibition enhanced pathways related to metabolism and proliferation, including glutathione metabolism and DNA replication. These findings suggest that *CCR2* signaling may maintain chronic inflammatory responses and immune dysregulation, while its inhibition promotes the transition from immune activation to cellular recovery and homeostasis.

According to some recent research, M2 macrophages are crucial for immunoregulatory processes in MTB infection, which may help the virus survive and evade the immune system (28–30). A study on patients with smoking-related pulmonary tuberculosis discovered that the proportion of M2 macrophages in the bronchoalveolar lavage fluid of pulmonary tuberculosis-smokers was significantly increased, accompanied by upregulation of inflammatory factors such as *MMP9* and *MMP12*. This suggests that MTB infection may enhance M2 polarization and promote immunosuppression in the context of smoking-related chronic inflammation (31). Additionally, MTB heat shock protein Hsp16.3 has been shown to induce macrophages to express M2 markers such as Arg-1, IL-10, and CD206, and its mechanism depends on CCRL2 and CX3CR1 receptors, activating the AKT/ERK/p38-MAPK signaling pathway (32). Another study has shown that Rocaglates, which are immunomodulators, can enhance macrophages’ response to IFN-γ and inhibit IL-4-induced M2 polarization, thereby promoting an M1-like phenotype and antibacterial ability. This suggests that reversing M2 polarization may be a key strategy for resisting chronic infection (33). Another study, from the perspective of the GRN/TNFR2 pathway, found that *GRN* can upregulate *TNFR2* expression and drive M2 polarization. After knocking out *GRN*, M1-related factors were upregulated, while M2 markers decreased, further confirming the key role of this axis in MTB-induced immunosuppression (17).

Studies have shown that *MCP-1* plays a key role in promoting macrophage polarization to the M2 phenotype. A study on hepatocellular carcinoma found that *SLFN11* deficiency can enhance the infiltration of immunosuppressive macrophages and promote their transformation to the M2-like phenotype by activating the Ccl2 signaling pathway, thereby exacerbating tumor progression (34). Another study also showed that nanofiber materials that continuously release *MCP-1* can induce macrophage polarization to the M2 type, which not only reduces foreign body reactions but also promotes angiogenesis (35). These studies together emphasize the core role of *MCP-1* in regulating the immunosuppressive microenvironment, especially in inducing M2 polarization. Consistent with these findings, our study further examined the regulatory function of MCP-1 in M2 macrophage polarization and immune suppression during MTB infection. This study further revealed the multiple mechanisms by which *MCP-1* regulates macrophage function in MTB infection. H37Ra stimulation significantly induced an increase in the proportion of CD206^+^ and CD163^+^ M2 macrophages, accompanied by upregulation of the expression of immunosuppressive factors ARG1 and PD-1. In contrast, MCP-1 knockdown effectively inhibited the expression of M2 marker molecules and slightly restored the level of M1 marker CD86. Besides, flow cytometry showed that *MCP-1* downregulation significantly enhanced the level of apoptosis induced by H37Ra. Analysis of inflammatory factors showed that MCP-1 downregulation promoted the release of TNF-α or IFN-γ and inhibited the secretion of TGF-β with IL-10, indicating that it maintained a balance between immunosuppression and pro-inflammatory responses. It is worth noting that MCP-1 also synergistically upregulated the expression of PD-1, TRIM30, and FAS immune regulatory factors, suggesting that it is involved in apoptosis, inflammatory regulation, and immune escape through the CCR2 pathway. In summary, MCP-1 is not only a key factor in the polarization of M2 macrophages induced by H37Ra but also plays a central role in regulating its anti-apoptotic and immunosuppressive functions.

This study has several limitations. Additional autophagy markers (e.g., LC3, p62) and host autophagy gene analyses were not performed, and MCP-1 effects were mainly studied in RAW264.7 cells without *in vivo* validation. The use of the attenuated H37Ra strain may limit generalizability to virulent Mtb infection, and the mechanistic link between autophagy modulation and M2 macrophage polarization remains to be established. Furthermore, a detailed characterization of H37Ra, including ESX system integrity and ESAT-6 production, was not conducted. These limitations highlight areas for further investigation in future studies to strengthen and extend the current findings.

## 5 Conclusion

This study revealed the multiple immune regulatory effects of the *CCR2/MCP-1* signaling axis in chronic MTB infection. *CCR2* antagonists can significantly reduce the expression of bacterial virulence factor ESAT-6, restore Beclin-1-mediated autophagy levels, reduce inflammatory damage in lung tissue, and improve tissue structure. Transcriptome analysis revealed *CCR2*-related immunosuppressive genes, including *TRIM30A*, *FAS*, and *PD-1*. Mechanistically, *MCP-1* promoted macrophage anti-apoptosis and induced M2 polarization, accompanied by upregulation of immunosuppressive cytokines (IL-10, TGF-β) and markers (CD206, CD163, ARG1). Moreover, knockdown of *MCP-1* reversed these effects and enhanced proinflammatory responses and autophagy. Together, these results collectively imply that the *CCR2/MCP-1* axis is involved in key immunoregulatory pathways in chronic MTB infection and might be a target for regulating the immune microenvironment of tuberculosis infection.

## Data Availability

The original contributions presented in the study are included in the article/supplementary material. Further inquiries can be directed to the corresponding authors.
